# Effectiveness of monetary incentives to recruit family physicians as study subjects: a randomized controlled trial

**DOI:** 10.1186/s13104-014-0969-8

**Published:** 2015-01-23

**Authors:** Anik MC Giguere, Michel Labrecque, Francine Borduas, Michel Rouleau

**Affiliations:** Office of Education and Continuing Professional Development, University Laval, Quebec, QC Canada; Department of Family and Emergency Medicine, Université Laval, Québec, QC Canada; Research Center of the CHU de Québec, Quebec, QC Canada; Department of Family and Emergency Medicine, Université Laval, Pavillon Ferdinand-Vandry, room 2881-C, 1050 avenue de la Médecine, Quebec, QC G1V 0A6 Canada

**Keywords:** Continuing medical education, e-learning, Compensation, Medical research methodology

## Abstract

**Background:**

Recruiting family physicians to participate as subjects of clinical studies is challenging. Monetary incentives are often used to increase enrolment, but few studies have measured the impact of doing so. As part of a trial seeking to compare two formats of interactive activities within an online continuing medical education (CME) program, we compared family physicians’ recruitment rates with and without a monetary incentive. Recruitment took place by email.

**Methods:**

Family physicians listed in the directory of the College of Physicians of the Province of Quebec (Canada) were emailed a one-page letter inviting them to participate in a randomized trial designed to evaluate a three-hour online CME program on rheumatology. Half of physicians were randomly allocated to receive a version of the letter that offered them $300 to participate (incentive group); the other half was not offered compensation (no-incentive group).

**Results:**

A total of 1314 (91%) physicians had a valid email address as listed in the directory. The response rate was 7.5% (54/724) in the incentive group and 2.6% (19/724) in the no-incentive group (absolute difference [AD] 4.8%, 95% confidence interval [95% CI] = 2.6 – 7.2%; risk ratio [RR] 2.8, 95% CI = 1.7 - 4.7). Recruitment rates were 3.5% (25/724) in the incentive group and 0.6% (4/724) in the no-incentive group (AD 2.9%, 95% CI = 1.5 - 4.5%; RR 6.3, 95% CI = 2.2 - 17.9).

**Conclusions:**

Monetary incentives significantly increased recruitment, which nonetheless remained low. To reach recruitment targets, researchers are advised to plan for an extensive list of email contacts and to minimize restrictive eligibility criteria.

## Background

Recruiting physicians to participate in health services research is often challenging [1-3]. Low participation rates can prevent the completion of an otherwise well-conceived and well-designed project [4,5], limit the extent to which results can be generalized to the relevant population [6] and reduce statistical power. Reported barriers to clinician or entire practice participation to research include time constraints, lack of staff and training, worry about the impact on the doctor-patient relationship, concern for patients, loss of professional autonomy, difficulty with the consent procedure, lack of rewards and recognition, and an insufficiently interesting question [7]. Common strategies for improving recruitment of clinicians include capitalizing on personal contacts and friendship networks, the use of physician recruiter, involvement of a local champion, minimization of the participation burden on the practice, and sizable incentives [1,3,8].

Systematic reviews of randomized trials have shown that offering physicians monetary incentives improves survey response rates [9] and patient recruitment [10]. In addition to improving recruitment, monetary incentives can be offered to influence the delivery of healthcare. They are generally effective in improving processes of care, referrals and admissions, and generally ineffective in improving compliance with guidelines outcomes [11]. Often, their impact is not sustained after the incentive is withdrawn [12].

To the best of our knowledge, no randomized trials have assessed how offering physicians monetary incentives affects physicians’ recruitment as subjects of an experimental study.

Our objective was to assess the impact on the recruitment rate of including an offer of monetary compensation in emails inviting family physicians to participate as study subjects in a trial of an online continuing medical education (CME) program.

## Methods

Of the 9104 family physicians listed in the 2004–5 directory of the College of Physicians of the Province of Quebec, Canada, 1448 identify an email address. The Office of Continuing Medical Education at Laval University in Quebec City, Canada, emailed all of these physicians, inviting them to participate in a study to evaluate two formats of an online CME program on rheumatology. The program promoted the integration of knowledge and skills on osteoarthritis and rheumatoid arthritis through practical and relevant case scenarios. The case studies were adapted from the ExpertMD™ program on rheumatology by two family physicians and were validated by a rheumatologist. The educational program was provided through synchronous or asynchronous interaction modes. The letters of invitation were one page long. Each letter was attached to the email as a separate document and was pasted in the body of the email itself.

All letters of invitation indicated that 1) the study was conducted by Laval’s Office of Continuing Medical Education in collaboration with the Faculty of Administration and was funded by an unrestricted grant from a pharmaceutical company; 2) participation consisted of one 3-hour session, but that to be eligible, physicians had to be available on the evenings of February 8 and February 9, 2005; 3) CME credits would be granted upon completion of the study; and 4) participants might be invited to participate in a second CME session. The invitation cited an email address from which physicians could obtain further information and to which they could respond to the invitation to participate. The letter was signed by the director of the Office of Continuing Medical Education.

Half of all physicians with an email address (n = 724) were randomly assigned to receive this letter of invitation. This population constituted the control (no-incentive) group. The other half were randomly assigned to receive a letter that was identical to the first except that it also offered a $300 incentive to participate in the study ($100/hour for 3 hours of participation). This population constituted the experimental (incentive) group. A repeat invitation was sent to all physicians who did not respond after a week. All physicians were randomly allocated to either group simultaneously using free online software (http://www.randomizer.org/), ensuring allocation concealment.

The Office of Continuing Medical Education at Laval University received and collated physicians’ responses to the invitation. Physicians’ response and recruitment rates were calculated as proportions of the number of physicians who were contacted at the onset of the study. We compared response rates based on intention-to-treat principles using the classical Normal-theory unadjusted confidence interval for the difference of 2 proportions. We also computed confidence interval for the relative risk using standard Normal approximation formulas. The project was approved by the Research Ethics Committee at Laval University.

## Results

**Figure 1:**
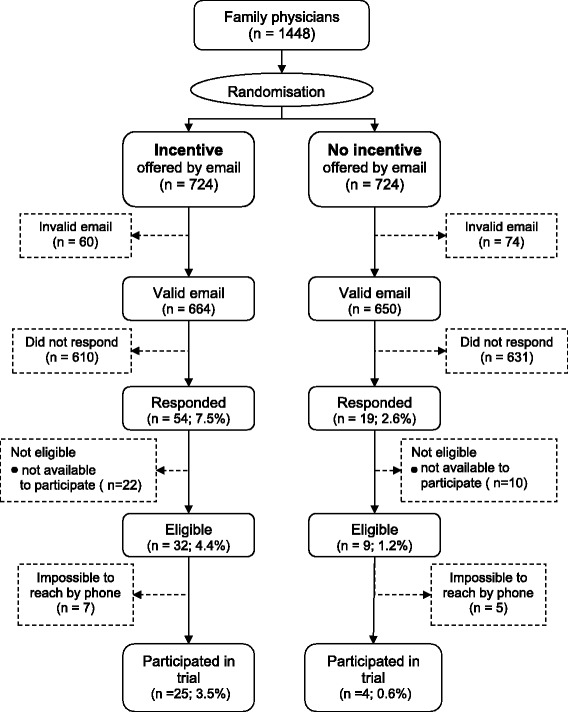
**Trial Flow Chart.**

Figure 1 presents the trial flow. A total of 1314 (91%) of physicians had a valid email address as listed in the directory. Of all physicians (both those allocated to the no-incentive group and those allocated to the incentive group), 73 (5.0%, 95% confidence interval [95% CI] = 4.0%–6.3%) responded to the invitation, either accepting, declining or requesting further information. Finally, 29 (2.0%, 95% CI = 1.4%–2.9%] were recruited to participate in the trial.

Physicians’ response rate was significantly higher in the incentive group than in the no-incentive group (7.5% versus 2.6%; absolute difference [AD] 4.8%, 95% CI = 2.6%–7.2%; risk ratio [RR] 2.8, 95% CI = 1.7–4.7). The recruitment rate was also higher in the incentive group (3.5% versus 0.6%; AD 2.9%, 95% CI = 1.5% – 4.5%; RR 6.3, 95% CI = 2.2 – 17.9).

## Discussion

Offering family physicians a monetary incentive to participate in a research project to evaluate an online CME program in rheumatology as part of an email invitation significantly increased physicians’ response and recruitment rates. It tripled the proportion of physicians responding to the email and multiplied the number of those recruited as study subjects by six.

Despite the effectiveness of our offering a monetary incentive, the response and recruitment rates of our recruitment strategy remained low, albeit similar to rates reported elsewhere. Kemper et al. [13] sent 29,000 emails inviting individuals to evaluate an Internet-based educational program, but only enrolled 1267 (4%) people. Cabana et al. [14] observed that primary care providers’ participation in the evaluation of an interactive asthma educational program was lowest when recruitment took place exclusively by email (10%); recruitment increased to 23% when the researchers added strategies such as face-to-face meetings and telephone calls.

Although our study design—a randomized trial—was robust, our study’s external validity is limited. First, physicians were recruited for a very specific purpose: participating as subjects in an experimental study of a CME program. Other purposes for recruitment could produce different results.

Second, our invitation letter clearly informed physicians that to participate, they had to be available on two consecutive evenings. This restriction was necessary due to a lack of availability of the expert who delivered training on any other date, but it may have decreased response rates. Less restrictive eligibility criteria may increase responses and recruitment.

Third, the relationship between the amount of the monetary incentive and recruitment is unclear. It is true that the amount offered was both substantial and commensurate with the task and with physicians’ time, but we found only two studies that addressed the effects of different amounts of remuneration. Ash et al. [8] observed that even substantial incentives ($250) do not guarantee high participation rates when recruiting community-based physicians for health services research, but none of the studies covered by Ash’s review experimentally tested the impact of incentives on recruitment. And in a systematic review of methods to increase questionnaire response rates, Edwards et al^4^ observed that compared to smaller incentives, larger incentives improved the odds that health providers would respond to postal questionnaires, but did not improve the odds that they would respond to electronic questionnaires.

Recruitment activities comprise successive steps to (1) gain entry at a clinical site, (2) obtain clinicians’ records to evaluate eligibility, (3) reach participants, (4) assess their willingness to participate and (5) schedule participation [15]. As monetary incentives act mostly to improve clinicians’ willingness to participate, the low recruitment rates observed likely reflect barriers at a prior step, here to reach participants. When planning a study, researchers are thus advised to plan carefully each of the critical steps, and use monetary incentives to improve physicians’ willingness to participate.

## Conclusions

Offering family physicians a monetary incentive as part of an email invitation to participate in an RCT designed to evaluate an online CME program significantly increased recruitment, which nonetheless remained low. To reach recruitment targets, researchers are advised to plan for an extensive list of email contacts and to minimize restrictive eligibility criteria.
